# Main and epistatic QTL analyses for Sclerotinia Head Rot resistance in sunflower

**DOI:** 10.1371/journal.pone.0189859

**Published:** 2017-12-20

**Authors:** Jeremías Enrique Zubrzycki, Carla Andrea Maringolo, Carla Valeria Filippi, Facundo José Quiróz, Verónica Nishinakamasu, Andrea Fabiana Puebla, Julio A. Di Rienzo, Alberto Escande, Verónica Viviana Lia, Ruth Amalia Heinz, Horacio Esteban Hopp, Gerardo D. L. Cervigni, Norma Beatriz Paniego

**Affiliations:** 1 Instituto de Biotecnología, Centro de Investigaciones en Ciencias Agronómicas y Veterinarias, Instituto Nacional de Tecnología Agropecuaria, Hurlingham, Buenos Aires, Argentina; 2 Laboratorio de Patología Vegetal, Unidad Integrada Universidad Nacional de Mar del Plata, Estación Experimental Agropecuaria INTA Balcarce, Balcarce, Buenos Aires, Argentina; 3 Consejo Nacional de Investigaciones Científicas y Técnicas, Ciudad Autónoma de Buenos Aires, Argentina; 4 Cátedra de Estadística y Biometría, Facultad de Ciencias Agropecuarias, Universidad Nacional de Córdoba, Córdoba, Argentina; 5 Facultad de Ciencias Exactas y Naturales, Universidad de Buenos Aires, Ciudad Autónoma de Buenos Aires, Argentina; 6 Centro de Estudios Fotosintéticos y Bioquímicos, Rosario, Santa Fe, Argentina; Nanjing Forestry University, CHINA

## Abstract

Sclerotinia Head Rot (SHR), a disease caused by *Sclerotinia sclerotiorum*, is one of the most limiting factors in sunflower production. In this study, we identified genomic loci associated with resistance to SHR to support the development of assisted breeding strategies. We genotyped 114 Recombinant Inbred Lines (RILs) along with their parental lines (PAC2 –partially resistant–and RHA266 –susceptible–) by using a 384 single nucleotide polymorphism (SNP) Illumina Oligo Pool Assay to saturate a sunflower genetic map. Subsequently, we tested these lines for SHR resistance using assisted inoculations with *S*. *sclerotiorum* ascospores. We also conducted a randomized complete-block assays with three replicates to visually score disease incidence (DI), disease severity (DS), disease intensity (DInt) and incubation period (IP) through four field trials (2010–2014). We finally assessed main effect quantitative trait *loci* (M-QTLs) and epistatic QTLs (E-QTLs) by composite interval mapping (CIM) and mixed-model-based composite interval mapping (MCIM), respectively. As a result of this study, the improved map incorporates 61 new SNPs over candidate genes. We detected a broad range of narrow sense heritability (*h*^*2*^) values (1.86–59.9%) as well as 36 M-QTLs and 13 E-QTLs along 14 linkage groups (LGs). On LG1, LG10, and LG15, we repeatedly detected QTLs across field trials; which emphasizes their putative effectiveness against SHR. In all selected variables, most of the identified QTLs showed high determination coefficients, associated with moderate to high heritability values. Using markers shared with previous Sclerotinia resistance studies, we compared the QTL locations in LG1, LG2, LG8, LG10, LG11, LG15 and LG16. This study constitutes the largest report of QTLs for SHR resistance in sunflower. Further studies focusing on the regions in LG1, LG10, and LG15 harboring the detected QTLs are necessary to identify causal alleles and contribute to unraveling the complex genetic basis governing the resistance.

## Introduction

*Sclerotinia sclerotiorum* (Lib.) de Bary is a widespread fungal pathogen in sunflower regions of the world [1]. It produces head rot, stalk rot, and wilt, among other diseases. Sclerotinia Head Rot (SHR) is one of the most common and damaging diseases in Argentina, Canada, China, Europe, South Africa and the United States. This disease accounts for 10 to 20% of average yield reduction in sunflower production, while in a season where humidity and temperature are both favorable for disease progression, SHR can produce the loss of the entire harvest [2].

Integrated management programs have been proposed to control *S*. *sclerotiorum* diseases in sunflower, including biological, chemical and physical control methods. However, total immunity has not been found in sunflower, and all improvement strategies rely on genomic tools such as molecular markers [3]. In this regard, the genetic basis of SHR resistance has been described as consisting of many genes of small quantitative effects whose expressions are highly dependent on the environment with different mechanisms involved in resistance at each phase of the disease in sunflower [4].

Sunflower genotyping tools have evolved over the years from restriction fragment length polymorphisms (RFLP), amplified fragment length polymorphism (AFLP), simple sequence repeat (SSR), sequence-tagged-site (STS) to single nucleotide polymorphism (SNP). Several genetic linkage maps have been published in the last 20 years [5–10]. Also, recent reports describe the development of medium to high throughput SNP panels and highly dense genetic maps [11–16].

Multiple traits (such as disease incidence, incubation period, lesion size, etc.) can be used to characterize quantitative trait *loci* (QTL) in SHR. A quantitative feature that can be reliably measured is essential for QTL mapping accuracy [17].

Several groups have studied QTLs for SHR resistance on diverse biparental populations using assisted ascospore inoculations [18–21]. QTLs have been reported on different linkage groups (LGs), corroborating the polygenic nature of inheritance. In most cases, the identified QTLs explains only a low percentage of phenotypic variation, within a range of 23% [21] to 44% [22]. Comparisons of QTLs among studies were limited because of the lack of shared markers and sequence information associated with the QTLs.

In recent years, the decrease in genotyping costs has motivated the development of new breeding strategies, which complements the classical QTL mapping techniques. Therefore, it is interesting to compare QTLs found through biparental mapping with those genomic regions associated with trait variations identified by association mapping (AM) [23,24]. In this regard, Fusari *et al*. [25] reported for the first time the use of AM to study SHR in inbred lines of sunflower from the breeding program of the National Institute of Agricultural Technology (INTA, Argentina) by using a candidate gene approach. They identified a significant association of the candidate gene *HaRIC_B* with SHR incidence. In addition, Talukder *et al*. [14] identified two candidate genes (*HaCOI1-1* and *HaCOI1-2*) that were significantly associated with Sclerotinia basal stalk rot resistance and that explained 7.4% of phenotypic variation.

In the research that we report here, we developed a new saturated AFLP-SSR-SNP linkage map for a population of sunflower recombinant inbreed lines derived (RILs) from a cross between PAC2 (partially resistant parental line) and RHA266 (susceptible parental line). We assessed the disease resistance level during the growing seasons of 2010 to 2014. Finally, the main-effect QTLs (M-QTLs), epistatic QTLs (E-QTLs) and the QTL × Environment interaction for the resistance traits disease incidence (DI), disease severity (DS), disease intensity (DInt), and incubation period (IP) were analyzed.

## Materials and methods

### Sunflower genotypes

A biparental mapping population (BMP) of 114 sunflower recombinant inbred lines (RILs) and the corresponding parental lines were used in the study. The BMP, and the parental lines, partially resistant PAC2 (P_R_) and the susceptible RHA266 (P_S_) [26], were provided by Dr. Gentzbittel and Dr. Langlade, from Institut National de la Recherche Agronomique (INRA, France).

### Disease assessment

Four field trials (FT) were carried out at the INTA Balcarce Experimental Station (Province of Buenos Aires, Argentina). The RILs and the two parental lines were sown in four growing seasons using a randomized complete block design with three replicates. The different trials were identified as FT1 (sowing date October 19^th^, 2009); FT2 (sowing date December 5^th^, 2011); FT3 (sowing date December 5^th^, 2012) and FT4 (sowing date December 9^th^, 2013). The experimental unit consisted of one row (10 m long and 0.7 m width) with 25 plants.

*S*. *sclerotiorum* ascospores were obtained from apothecia developed by inducing carpogenic germination of sclerotia, as described by Escande *et al*. [27]. Individual capitula at R5.2—R5.5 stage [28] were directly inoculated by mechanical spray with 1 ml of a suspension containing 2,500 ascospores ml^-1^. After inoculation, the capitula were covered with paper bags for 10 days [29]. The capitula were spray-irrigated, daily at noon for 20 min, to maintain humid conditions until physiological maturity. A susceptible cultivar was simultaneously inoculated with the population at every inoculation date to check the efficacy of the inoculation procedure.

### Phenotypic evaluation and statistical analysis

The phenotypic evaluation was carried out on individual capitula. SHR measures were recorded as disease incidence (DI), disease severity (DS), disease intensity (DInt), and incubation period (IP) in each plot. DI was registered as the maximum measured ratio of symptomatic capitula among the total number of inoculated plants per plot, DS as the average proportion of the rotted receptacle area of all diseased capitula at 21 DPIs, DInt as the average proportion of rotted receptacle area of all inoculated capitula at 21 DPIs, and IP as the average DPIs in which the symptoms appear of all symptomatic capitula assessed at 10, 14, 17, 19, 21, 24 and 28 DPIs.

To avoid inter-rater errors, the same observer conducted all assessments.

A generalized mixed linear model (GMLM) or a linear mixed-effects model (LMEM) was fitted for each disease variable depending on the variable distribution and according to Filippi *et al*. [30].

DI value, as the number of diseased plants over a total of inoculated plants, follows a binomial distribution. It was analyzed according to the following model:

$$\text{log}\;\left(\frac{\pi_{ijkl}}{1-\pi_{ijkl}}\right)\;=\;\mu +\;\lambda_{i}+\;c_{j}+f_{kj}+\;\lambda f_{ikj}+b_{lj}$$ (1)

where *π*_*ijkl*_ represents the probability of a plant becoming infected if it belongs to the inbred line *i* (λ_*i*_), evaluated in the field trial *j* (*c*_*j*_), inoculated in date *k* at the field trial *j* (*f*_*kj*_), and located in block *l* at the field trial *j* (*b*_*lj*_). The term *λf*_*ikj*_ refers to the interaction between line and date of inoculation.

DS, DInt, and IP were analyzed using LMEM, after verifying normality, according to the following model:

$$Y_{ijkl}\;=\;\mu +\;\lambda_{i}+\;c_{j}+f_{kj}+\;\lambda f_{ikj}+{b{}_{lj}+e}_{ljkl}$$ (2)

where ***Y***_*ijkl*_ represents the disease measure for the inbred line *i* (λ_*i*_), evaluated in the field trial *j* (*c*_*j*_), inoculated in date *k* at the field trial *j* (*f*_*kj*_), and located in block *l* at the field trial *j* (*b*_*lj*_). The term *λf*_*ikj*_ refers to the interaction between line and date of inoculation, and ***ε***_*ijkl*_ is the normal error term for the observation ***Y***_*ijkl*_.

The inclusion of the line date-of-inoculation interaction term in models (1) and (2) is due to the broad diversity in flowering time among the RILs. To include this term we take care that effects of line and flowering time were not confounded.

Pearson correlation coefficients (r) were calculated from adjusted means to evaluate the associations of SHR traits. Narrow sense heritability (*h*^*2*^) was estimated for each trial by introducing the “inbred line” as a random effect in models (1) and (2). Thus, the percentage of genotypic contribution to total phenotypic variation explained (PVE) was used to estimate the corresponding heritability [31]. All the statistical analyses were conducted using InfoStat statistical software [32], which uses nlme [33] and lme4 [34] R packages [35] for model fitting.

### DNA preparation and SNP genotyping

Total genomic DNA was extracted from 20 mg of 3-weeks-old lyophilized leaves collected from 114 RILs and both parental lines with the NucleoSpin Plant II system (Macherey-Nagel, Düren, Alemania). DNA quality was assessed using electrophoretic analysis, while DNA concentration was determined with spectrofluorometer Nanodrop ND 3300 (NanoDrop Technologies, Wilmington, DE, US) and the Quant-iT PicoGreen dsDNA reagent (Invitrogen, Carlsbad, CA, US). Genomic DNA was normalized to 50 ng/μl.

Genotyping was performed using a custom-designed 384 Illumina Sunflower Oligo Pool Assay (SOPA) [36,37]. The SOPA design was based on the sunflower unigene collection [38]. Briefly, *in silico* SNP markers were identified using the CAP3 assembly outputs as the input of an in-house pipeline that finds biallelic positions in sequence alignments of Expressed Sequence Tag (EST) markers. The selection criteria applied for SNP calling was at least two distinct alleles in EST alignments with a minimum of five sequences, and no flanking polymorphism (50 bp up/downstream). Additional sequences of pre-validated SNPs from Fusari *et al*. [25,39] and Kolkman *et al*. [40] were included in the SOPA design process. A total of 2,867 sequences containing SNPs were submitted to the Assay Design Tool (ADT, Illumina, San Diego, CA, US), that uses information on the flanking sequences to identify loci with high likelihood of success [41]. SNPs with ADT score ≥ 0.6 and a designability score ranging 0.5 to 1 were pre-selected. Finally, the last filter applied for the selection of 384 SNPs was the functional annotation assigned to each unigene [38] (atgc-sur.inta.gob.ar, choosing those related to biotic and abiotic stress).

Genotyping was performed on an Illumina, BeadXpress reader (Illumina, San Diego, CA, US) at the Biotechnology Institute of INTA Castelar (Province of Buenos Aires, Argentina) using the manufacturer’s protocol [41]. To evaluate the assay reproducibility, we included in each plate eight replicated samples of P_R_, seven replicated samples of P_S_ and negative controls. SNP intensities per sample were normalized and assigned to a cluster position. According to Illumina recommendation, the quality value for samples, i.e., "call rate" was > 0.70. Samples below this value were discarded. A GenCall cutoff of 0.25 and a value < 0.40 for a GenTrain Score were set to determine reliable allele calling at each SNP, following the recommendations from previous studies [42,43].

When automated cluster separation was not apparent (i.e. presenting overlapping clusters, unassigned samples, and apparently heterozygous genotypes), clusters were manually edited to identify SNPs as polymorphic or monomorphic unambiguously.

### Genetic map construction

Informative SNPs were incorporated into a previous genetic mapping matrix [10,44,45]. SNP segregation in the progeny was assessed with the program GQMOL [46] to identify significant deviations (*p* ≤ 0.05) of observed frequencies from the expected Mendelian segregation using False Discovery Rate (FDR) correction [47].

The genetic map was constructed using the software package JoinMap, version 3.0 [48]. Marker grouping was performed using likelihood odds (LOD) ratios with a LOD threshold of 4.0 and a maximum recombination fraction threshold of 0.35 [49]. The order obtained in previous mapping efforts was kept unchanged for map calculations of the groups by specifying a starting order in the corresponding JoinMap tabsheet. Similarly, the remaining ungrouped *loci* were then assigned to LGs according to previously published genetic maps by using the “move selected *loci*” function from the “Grouping” menu reducing LOD stringency to 1.0.

Recombination frequencies were transformed into centiMorgans (cM) by using Kosambi’s mapping function [50] and corrected by the factor that considers multiple generations of meiosis in RIL populations [7,51].

Finally, the LGs were plotted with the software Mapchart 2.2 [52].

### QTL mapping

For QTL mapping, we used the adjusted means per RILs and the built genetic map. Single-locus QTL mapping was performed using the software QTL Cartographer 2.5 [53] applying the method of composite interval mapping (CIM) with cofactors [54] to detect, map and characterize the main effect QTLs (M-QTLs). “Method 6” and a maximum of five markers were automatically selected as cofactors over a window size of 10 cM by the forward and backward regression method with both, probabilities-to-enter and to-delete = 0.1. The chosen walk speed was 0.5 cM. A LOD score threshold to declare a putative QTL as significant was chosen by performing 1000 permutations for α = 0.05 [55]. QTL position was determined by the maximum LOD value within the region under analysis while confidence intervals were obtained using positions ± 1 LOD away from the peak. PVE by a given QTL was estimated by its determination coefficient (*R*^*2*^).

QTL Cartographer output file was employed to conduct two-locus analysis with QTLNetwork 2.1 [56], which is performed on mixed-model-based composite interval mapping (MCIM) [57]. This program identifies epistatic QTLs (Ep-QTLs) and M-QTLs. Critical F-values were calculated through a 1000 permutation test. M-QTLs and Ep-QTLs were declared as putative with a significance level of 0.05. A Monte Carlo Markov Chain approach was used to estimate QTL effects.

QTLs detected here were generically designated as “*qVARIABLE-LG”* [58], for genomic regions detected with both QTL Cartographer and QTLNetwork. A letter was added at the end of the name for differentiating all the QTLs found in the same LG. QTLs and confidence intervals were plotted on the genetic map with the software Mapchart 2.2 [52].

Parent allele contributions to resistance were evaluated by taking only locus-specific markers near maximum LOD values (defining M-QTLs). Therefore, AFLP markers (names starting with “E”) were not considered. Finally, maps from previous QTL analysis for Sclerotinia resistance in sunflower were cross-referenced with the current map through shared markers to compare the chromosomal positions of M-QTLs from different studies.

## Results

### Statistical analysis of SHR-related phenotypic variables

Table 1 displays the general statistics for the four SHR-related phenotypic variables. We analyzed the selected phenotypic variables of 99 RILs of a total of 114. We discarded the remaining 15 RILs because we were unable to obtain phenotypic data mainly due to low seed germination.

**Table 1:** Statistical analysis for four disease resistant traits of RIL population and parents in multiple environments.

Trait	Field Trial ID^a^	Biparental Mapping Population							*h*^*2*^ (%)
		Min	Max	Mean	SD	Skewness	Kurtosis	CV (%)	
**DI**									
	FT1	0.00	1.00	0.51 ± 0.02	0.27	-0.17	-0.95	52.95	45.61
	FT2	0.00	0.75	0.11 ± 0.01	0.18	1.78	2.26	161.56	7.34
	FT3	0.00	1.00	0.65 ± 0.02	0.25	-0.54	-0.24	37.97	34.98
	FT4	0.00	1.00	0.48 ± 0.02	0.29	0.09	-0.99	60.14	31.49
	AFT	0.00	1.00	0.45 ± 0.01	0.32	0.01	-1.23	70.67	N/A
**DS**									
	FT1	0.01	1.00	0.18 ± 0.01	0.14	3.18	13.52	79.40	20.40
	FT2	0.01	1.00	0.30 ± 0.03	0.27	0.90	-0.10	92.76	43.36
	FT3	0.01	1.00	0.51 ± 0.01	0.21	0.04	-0.23	41.08	20.49
	FT4	0.01	0.80	0.29 ± 0.02	0.18	0.27	-0.56	62.04	50.52
	AFT	0.01	1.00	0.35 ± 0.01	0.24	0.59	-0.37	68.64	N/A
**DInt**									
	FT1	0.00	0.36	0.04 ± 0.0038	0.05	3.90	20.14	131.87	1.86
	FT2	0.00	0.36	0.03 ± 0.0044	0.06	3.01	9.93	220.20	2.87
	FT3	0.00	0.88	0.28 ± 0.01	0.19	0.60	-0.31	67.99	26.49
	FT4	0.00	0.70	0.12 ± 0.01	0.14	1.41	1.75	112.52	59.90
	AFT	0.00	0.88	0.13 ± 0.01	0.17	1.56	1.89	130.24	N/A
**IP**									
	FT1	16.00	42.00	27.91 ± 0.31	4.43	-0.01	0.02	15.88	13.74
	FT2	14.00	28.00	20.26 ± 0.41	3.56	0.39	-0.51	17.57	45.48
	FT3	14.00	28.00	19.49 ± 0.19	2.75	0.32	-0.24	14.08	37.94
	FT4	14.00	28.00	20.91 ± 0.25	2.86	-0.12	-0.48	13.67	52.45
	AFT	14.00	42.00	22.63 ± 0.20	5.13	0.77	0.16	22.66	N/A

^a^Field Trial ID FT1-4 and AFT represent: Balcarce October 2009; Balcarce December 2011; Balcarce December 2012; Balcarce December 2013; Across Field Trials, respectively.

DI = Disease Incidence

DS = Disease Severity

DInt = Disease Intensity

IP = Incubation Period

SD = Standard Deviation

CV = Coefficient of variation

N/A = not assigned

The heritability (*h*^*2*^) values were moderate for all traits across trials, except for DI in FT2 and DInt in FT1 and FT2.

Correlations among the phenotypic adjusted means are presented in S1 Table. For example, DI and DInt showed an expected positive correlation. By contrast, the correlations between IP and DS (r = -0.67, *p* < 0.0001), IP and DInt (r = -0.64, *p* < 0.0001) and IP and DI (r = -0.45, *p* < 0.0001) were negatives. Finally, the correlations between DI and traits DS and DInt (r = 0.41 and r = 0.64, respectively, p < 0.0001) were moderate.

### SNP genotyping assay and genetic map construction

For the SOPA design, we selected a total of 384 loci, out of the 537 putative SNP that matched the optimal designability criteria suggested by Illumina. The dataset includes 97 SNP polymorphisms previously validated by re-sequencing different sunflower genotypes [25,39,40] and 287 *in silico* SNP coming from EST functionally annotated as related to stress response according to Gene Ontology (see atgc-sur.inta.gob.ar).

The SOPA was assayed using the BMP 114 RILs giving good quality scores for 108 RILs (call rate ≥ 0.8%). The genotyping assay reproducibility was 100% for all replicated samples. Sixty-four (17%) of SNP were polymorphic, 218 (57%) were monomorphic, and 102 (26%) failed. The conversion rate, corresponding to the number of polymorphic SNPs divided by the total number of SNPs in the assay, was 0.17 (64/384). A total of 64 SNPs was available for mapping.

No significant deviations from Mendelian segregation ratios were observed for SNP markers from BMP RILs. The 64 polymorphic SNPs were incorporated into a previous genetic mapping matrix, rendering a final matrix composed of 114 RILs and 706 markers.

**Fig 1:**
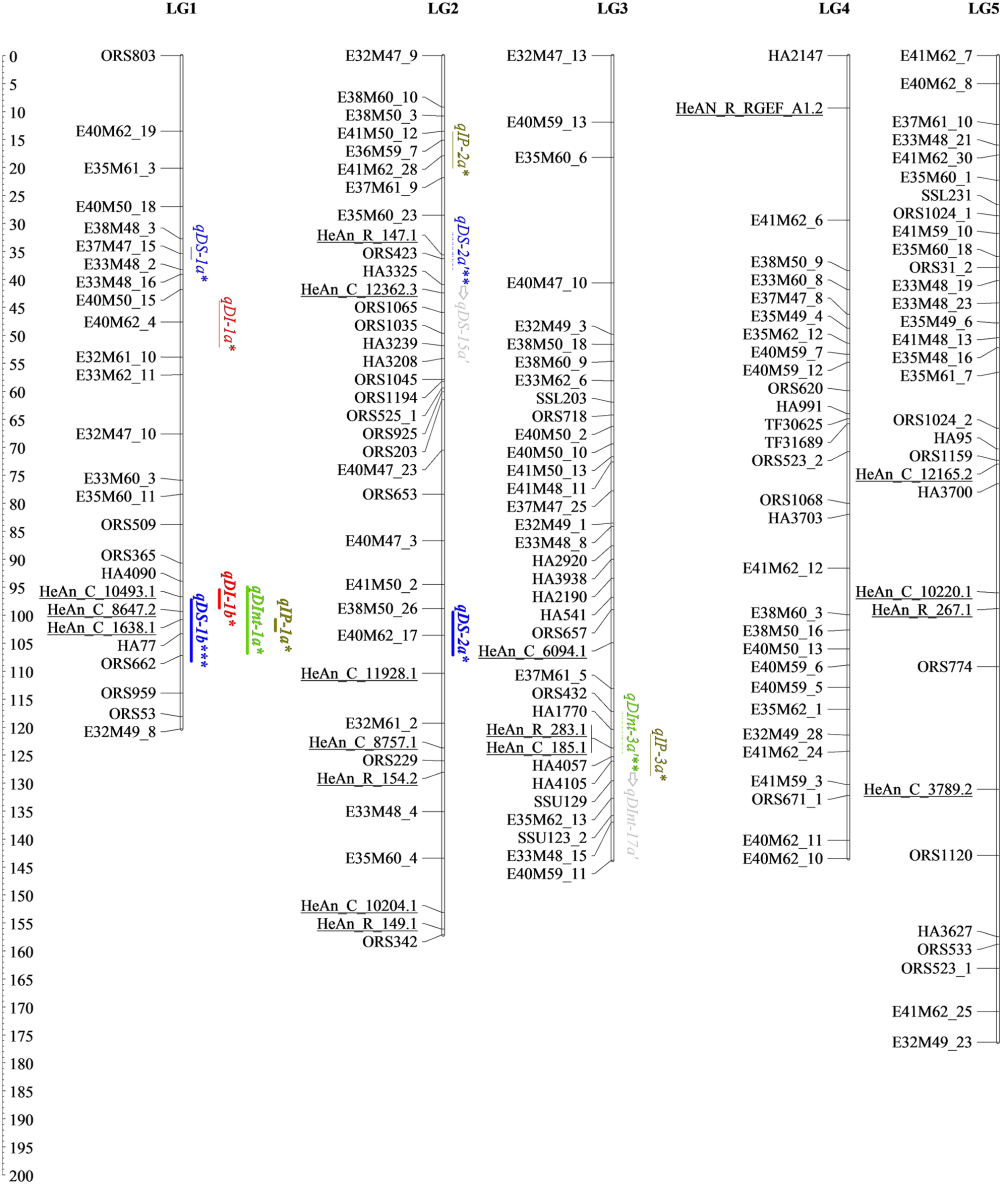
Genetic linkage map of BMP showing locations on LG1-5 of different types of QTLs and interactions detected through single- and two-locus QTL analyses for resistance traits. Mapped markers are listed on the left. Underlined markers correspond to the 61 SNPs mapped in this work. Lengths of confidence Interval (CI) for the M-QTL and the Ep-QTL are denoted by full lines and dashed lines, respectively. Epistatic interaction between QTLs is represented with arrows. SHR QTLs coincident with previous reported QTLs are in bold. *QTLs detected using QTL Cartographer. **QTLs detected using QTLNetwork. ***QTLs detected using both programs.

**Fig 2:**
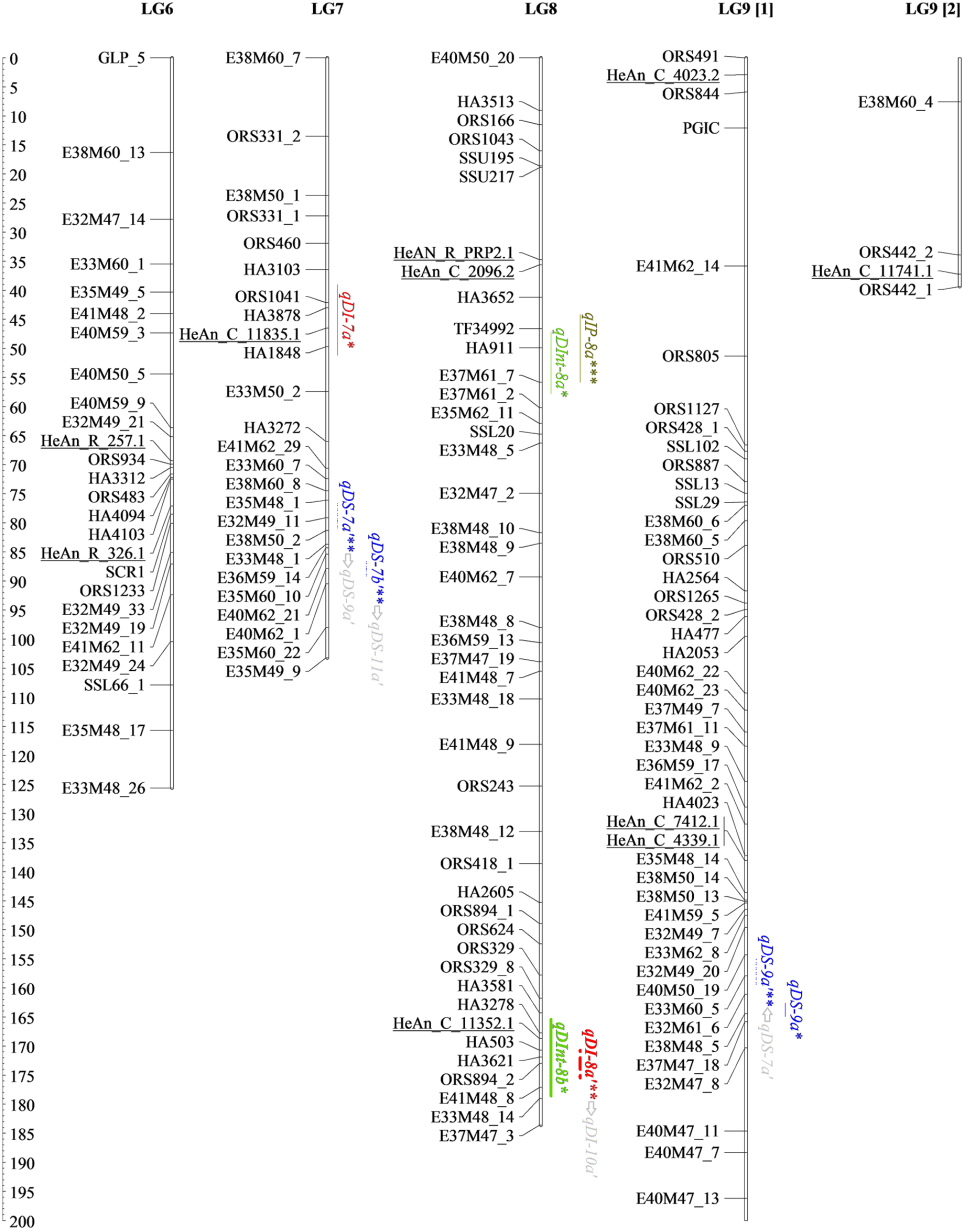
Genetic linkage map of BMP showing locations on LG6-9 of different types of QTLs and interactions detected through single- and two-locus QTL analyses for resistance traits. Mapped markers are listed on the left. Underlined markers correspond to the 61 SNPs mapped in this work. Lengths of confidence Interval (CI) for the M-QTL and the Ep-QTL are denoted by full lines and dashed lines, respectively. Epistatic interaction between QTLs is represented with arrows. SHR QTLs coincident with previous reported QTLs are in bold. * QTLs detected using QTL Cartographer. ** QTLs detected using QTLNetwork. *** QTLs detected using both programs.

**Fig 3:**
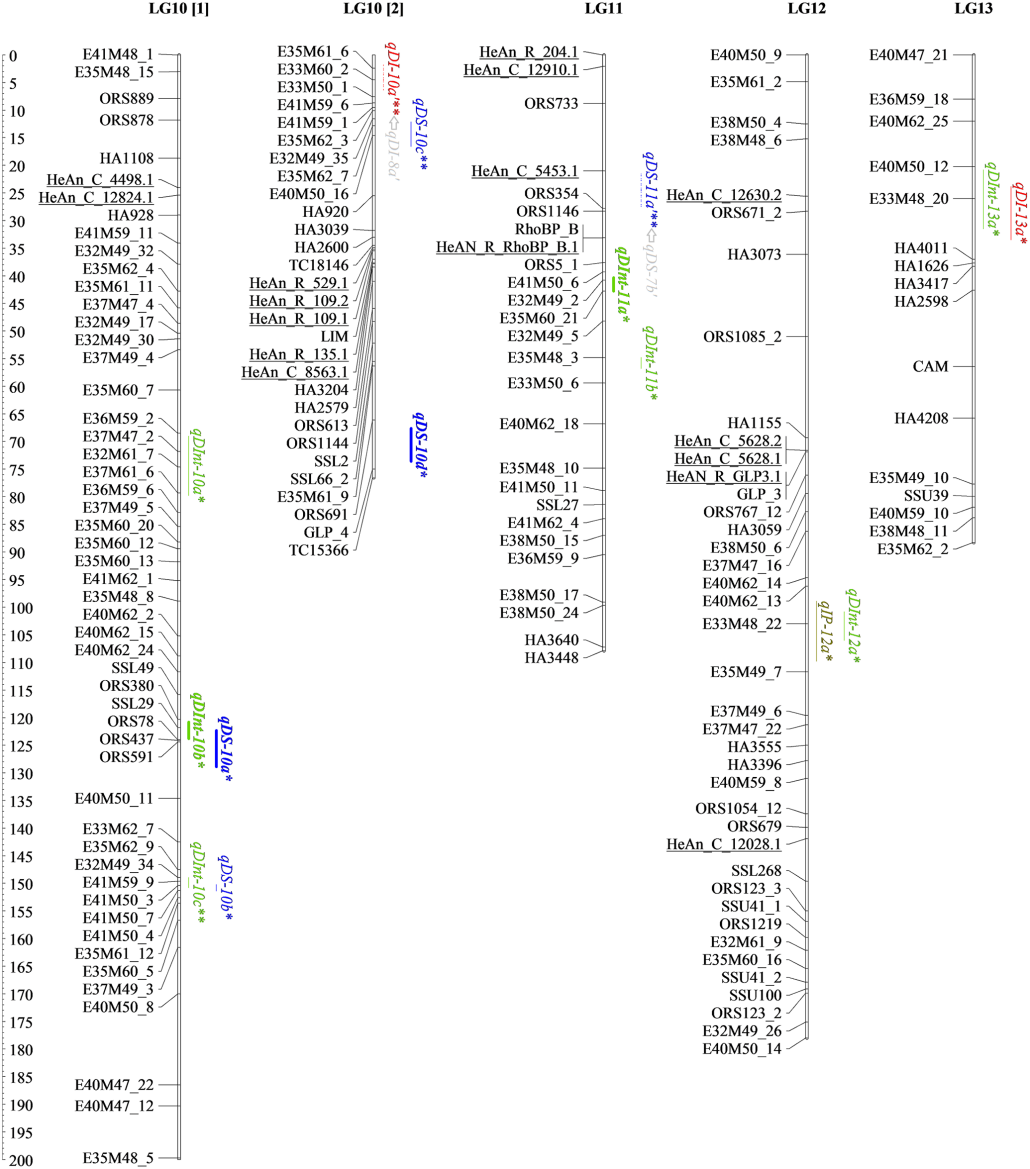
Genetic linkage map of BMP showing locations on LG10-13 of different types of QTLs and interactions detected through single- and two-locus QTL analyses for resistance traits. Mapped markers are listed on the left. Underlined markers correspond to the 61 SNPs mapped in this work. Lengths of confidence Interval (CI) for the M-QTL and the Ep-QTL are denoted by full lines and dashed lines, respectively. Epistatic interaction between QTLs is represented with arrows. SHR QTLs coincident with previous reported QTLs are in bold. * QTLs detected using QTL Cartographer. ** QTLs detected using QTLNetwork. *** QTLs detected using both programs.

**Fig 4:**
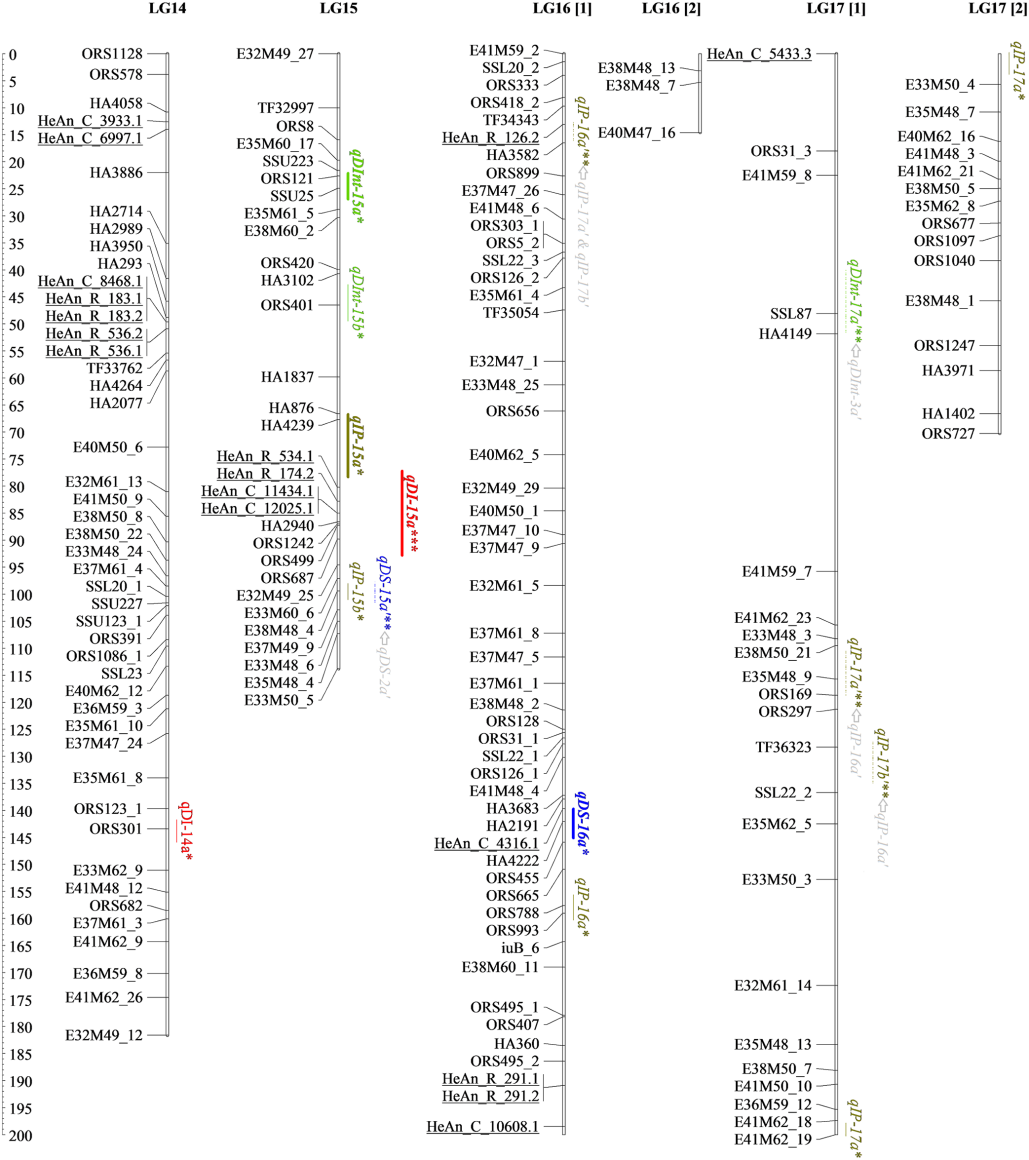
Genetic linkage map of BMP showing locations on LG14-17 of different types of QTLs and interactions detected through single- and two-locus QTL analyses for resistance traits. Mapped markers are listed on the left. Underlined markers correspond to the 61 SNPs mapped in this work. Lengths of confidence Interval (CI) for the M-QTL and the Ep-QTL are denoted by full lines and dashed lines, respectively. Epistatic interaction between QTLs is represented with arrows. SHR QTLs coincident with previous reported QTLs are in bold. * QTLs detected using QTL Cartographer. ** QTLs detected using QTLNetwork. *** QTLs detected using both programs.

The genetic map developed here for detecting SHR resistance QTLs contains 61 new SNPs from the SOPA (names starting with “HeAn”), eight SNPs from candidate genes, 327 AFLPs, 229 SSRs, eight EST-SSRs and two InDels (Figs 1–4 and Table 2). All *loci* were placed into 17 LGs, matching the expected chromosome number (n = 17). The map spanned 2,823.30 cM with an average density of one marker per 4.45 cM. The average size of LGs was 166.11 cM and ranged from 88.31 (LG13) to 276.64 cM (LG10), while the greatest length between two markers was 44 cM (LG17).

**Table 2:** Marker density and marker distribution on sunflower linkage groups (LGs).

LG	# AFLP	# SSR	# EST-SSR	# InDel	# SNP^a^	# HeAn^b^	# *loci*	Length (cM)	Density (cM/*locus*)
**1**	15	8	-	-	-	3	26	120.34	4.63
**2**	16	14	-	-	-	7	37	157.11	4.25
**3**	19	13	-	-	-	3	35	143.77	4.11
**4**	20	7	2	-	-	1	30	143.50	4.78
**5**	16	12	-	-	-	4	32	176.26	5.51
**6**	15	7	-	-	2	2	26	125.55	4.83
**7**	16	8	-	-	-	1	25	103.15	4.13
**8**	19	20	1	-	-	3	43	183.59	4.27
**9**	27	18	-	-	1	4	50	239.35	4.79
**10**	50	20	1	1	2	7	81	276.64	3.42
**11**	14	7	-	-	1	4	26	107.86	4.15
**12**	17	17	-	-	1	5	40	178.00	4.45
**13**	9	6	-	-	1	-	16	88.31	5.52
**14**	19	19	-	1	-	7	46	181.63	3.95
**15**	11	14	1	-	-	4	30	113.79	3.79
**16**	21	26	2	-	-	5	54	214.61	3.97
**17**	23	13	1	-	-	1	38	270.34	7.11
**Total**	327	229	8	2	8	61	635	2,823.80	-
**Mean**	19.24	13.47	0.47	0.12	0.47	3.59	37.35	166.11	4.45

^a^SNPs previously mapped [25,44–45]

^b^HeAn-SNPs identified and mapped in this work.

The S2 Table provides a detail of the mapped SNPs.

### Main effect QTLs for SHR phenotypic variables

A total of 36 M-QTLs (34 detected by single-locus analysis and five identified by two-locus analysis; three in common) were associated with SHR resistance. Figs 1–4, Tables 3 and 4 display detailed information about location, *R*^*2*^, and the additive effect of M-QTLs detected for SHR-related phenotypic variables. For DI, we found six M-QTLs located on LG1, LG7, LG13, LG14 and LG15 (six detected by single-locus analysis and one by two-locus analysis; one in common). *R*^*2*^ values ranged from low to moderate, explaining from 12.45% to 23.87% of the phenotypic variance. Resistance alleles came from the P_R_ except for *qDI-7a*, which came from P_S_. All but three M-QTLs (*qDI-13a*, *qDI-7a*, and *qDI-1a*) presented significant negative additive effects.

**Table 3:** Parameters associated with main effect QTLs detected in BMP by single-locus analysis using QTL Cartographer.

Trait	QTL	Field Trial^a^	LG	Marker^b^	Position on LG (cM)	LOD score	Additive effect (a)	% Phenotypic Variation Explained (*R*^*2*^)	Confidence interval (cM)	Parental contribution
**DI**										
	*qDI-1a*	FT3	1	E40M62_4	48.60	3.71	0.08	13.61	8.30	N/A
	*qDI-1b*	FT2	1	HeAn_C_10493.1	96.70	4.30	-0.04	15.15	3.30	P_R_
	*qDI-7a*	FT1	7	ORS1041	42.10	3.70	0.09	12.45	12.20	P_S_
	*qDI-13a*	AFT	13	E33M48_20	27.50	4.43	0.08	15.94	9.70	N/A
	*qDI-14a*	FT3	14	ORS301	143.40	4.11	-0.04	16.06	4.20	P_R_
	*qDI-15a*^c^	AFT	15	HeAn_R_534.1	80.20	7.25	-0.09	22.63	4.50	P_R_
		FT3	15	HeAn_C_12025.1/HeAn_C_11434.1	85.00	6.15	-0.10	19.77	4.00	P_R_
		FT4	15	ORS1242	87-0	4.10	-0.10	14.71	3.30	P_R_
		AFT	15	ORS687	90.80	6.87	-0.09	23.87	4.50	P_R_
**DS**										
	*qDS-1a*	FT1	1	E37M47_15	35.40	5.36	-0.04	24.92	2.20	N/A
	*qDS-1b*^c^	FT2	1	HeAn_C_8647.2	99.30	5.67	-0.19	29.12	11.00	P_R_
		FT2	1	HA77	104.80	4.63	-0.19	28.10	11.00	P_R_
	*qDS-2a*	AFT	2	E40M62_17	102.80	3.41	-0.05	12.05	7.70	N/A
	*qDS-9a*	FT4	9	E38M48_5	164.40	4.86	-0.07	20.84	2.30	N/A
	*qDS-10a*	AFT	10	ORS591	124.40	4.57	-0.06	16.97	6.60	P_R_
	*qDS-10b*	FT3	10	E41M50_3	150.40	4.22	-0.08	16.20	0.70	N/A
	*qDS-10d*	FT2	10	GLP_4	271.10	4.94	0.16	29.65	6.00	P_S_^NS^
	*qDS-16a*	FT1	16	HA4222	142.10	3.44	0.04	13.90	5.40	P_S_
**DInt**										
	*qDInt-1a*	FT2	1	HeAn_C_8647.2	98.70	4.25	-0.02	15.65	11.90	P_R_
		FT2	1	ORS662	106.80	4.52	-0.02	18.66	11.90	P_R_
	*qDInt-8a*	FT3	8	E37M61_7	54.50	4.00	-0.06	16.17	10.70	N/A
	*qDInt-8b*	FT4	8	HA3278	167.70	3.78	-0.05	14.76	13.20	P_R_
	*qDInt-10a*	FT3	10	E32M61_7	76.10	4.27	-0.07	20.14	10.80	N/A
	*qDInt-10b*	AFT	10	SSL39	121.80	3.81	-0.03	14.87	3.00	P_R_
	*qDInt-11a*	FT1	11	E32M49_2	41.30	3.73	0.02	15.23	2.50	N/A
	*qDInt-11b*	FT1	11	E35M48_3	54.80	4.25	-0.02	17.38	2.00	N/A
	*qDInt-12a*	FT1	12	E33M48_22	103.00	6.13	0.02	23.45	5.20	N/A
	*qDInt-13a*	FT2	13	E33M48_20	24.30	4.71	0.02	20.50	10.70	N/A
	*qDInt-15a*	FT1	15	SSU25	24.60	3.08	-0.01	12.05	4.80	P_R_^NS^
	*qDInt-15b*	FT1	15	ORS401	46.50	5.33	0.02	23.05	6.80	P_S_
**IP**										
	*qIP-1a*	AFT	1	HA77	102.30	7.35	1.25	29.05	2.10	P_R_
	*qIP-2a*	FT3	2	E41M62_28	17.90	4.64	-1.17	17.39	6.50	N/A
	*qIP-3a*	FT1	3	HeAn_R_283.1	123.70	4.23	1.27	16.76	7.50	P_R_
	*qIP-8a*^c^	FT2	8	HA911	50.00	4.26	1.86	21.92	5.10	P_R_
	*qIP-12a*	FT4	12	E33M48_22	105.00	3.65	-1.02	14.94	10.90	N/A
	*qIP-15a*	AFT	15	HA4239	71.70	4.26	1.02	19.95	11.60	P_R_
	*qIP-15b*	FT4	15	E38M48_4	99.40	5.35	1.17	20.70	3.00	N/A
	*qIP-16a*	AFT	16	ORS788	157.70	4.37	0.92	15.61	4.70	P_R_
	*qIP-17a*	FT1	17	E41M62_19	201.00	4.59	1.55	24.15	6.00	N/A

^a^FT1 = Balcarce October 2009, FT2 = Balcarce December 2011, FT3 = Balcarce December 2012, FT4 = Balcarce December 2013, AFT = across field trials, pooled data from all the trials.

^b^Marker defining QTL, closer to the highest LOD value.

^c^QTL detected by both QTL Cartographer and QTLNetwork.

DI = Disease Incidence

DS = Disease Severity

DInt = Disease Intensity

IP = Incubation Period

N/A = not assigned

**Table 4:** Parameters associated with main-effect QTLs and those involved in interaction with the trial (environment) detected in BMP by two-locus analysis using QTLNetwork.

Trait	QTL	LG	Marker^a^	Position on LG (cM)	Confidence interval (cM)	Additive effect (a)	*h*^*2*^a (%)^b^	QTL × Environment interactions		
								Field Trial^c^	Additive effects (ae)	*h*^*2*^ae (%)^d^
**DI**										
	*qDI-15a*^e^	15	HeAn_R_534.1	80.70	8.00	-0.08	15.32	-	-	-
**DS**										
	*qDS-1b*^e^	1	HeAn_C_8647.2	99.30	2.60	-0.06	7.43	FT1, FT2	-0.09 to 0.05	1.22 to 4.63
	*qDS-10c*	10	E40M50_16	214.30	4.50	-0.01	0.08	FT2, FT3	-0.04 to 0.06	1.22 to 2.81
**DInt**										
	*qDInt-10c*	10	E41M50_3	150.30	1.90	-0.03	6.20	FT1, FT2, FT3	-0.03 to 0.02	1.56 to 2.22
**IP**										
	*qIP-8a*^e^	8	HA911	51.90	8.60	0.88	9.81	-	-	-

^a^Marker defining QTL, closer to the highest LOD value.

^b^Percentages of the phenotypic variations explained by additive effects.

^c^Field trial in which Q × E was detected for the particular QTL. FT1 = Balcarce October 2009, FT2 = Balcarce December 2011, FT3 = Balcarce December 2012, FT4 = Balcarce December 2013, AFT = across field trials, pooled data from all the trials.

^d^Percentage of the phenotypic variations explained by the additive effect of the QTL × environment interaction.

^e^QTL detected by both QTL Cartographer and QTLNetwork.

DI = Disease Incidence

DS = Disease Severity

DInt = Disease Intensity

IP = Incubation Period

In this work, we identified nine M-QTLs for DS located on LG1, LG2, LG9, LG10 and LG16, eight detected by single-locus analysis and two by two-locus analysis; one shared between both approaches. For this variable, the range of *R*^*2*^ values was the widest observed across all analyzed variables, ranging from 0.08% to 29.65%. P_R_ provided resistance alleles for all M-QTLs detected for DS, except for *qDS-16a*. All but two M-QTLs, *qDS-10d* and *qDS-16a*, presented significant negative additive effects.

For DInt, we found 12 M-QTLs located on LG1, LG8, LG10, LG11, LG12, LG13 and LG15, 11 detected by single-locus analysis and one by two-locus analysis; none in common. *R*^*2*^ values ranged from 6.20% to 23.45%. Resistance alleles came from the P_R_ except for *qDInt-15b* that came from P_S_. All but four M-QTLs, *qDInt-11a*, *qDInt-12a*, *qDInt-13a*, and *qDInt-15b*, showed significant negative additive effects.

For IP, nine M-QTLs located on LG1, LG2, LG3, LG8, LG12, LG15, LG16 and LG17, nine detected by single-locus analysis and one by two-locus analysis; one in common, were identified. *R*^*2*^ values ranged from 9.81% to 29.05%. Resistance alleles came from the P_R_ and all but two M-QTLs, *qIP-2a* and *qIP-12a*, presented significant positive additive effects.

Interestingly, over the distal region of LG1, three markers from the SOPA, HeAn_C_10493.1, HeAn_C_8647.2 and HeAn_C_1638.1, were statistically associated with four M-QTLs, one for each tested variable, *qDI-1b*, *qDS-1b*, *qDInt-1a* and *qIP-1a*, being three of them consistently detected for FT2. Additionally, since *qDS-1b* and *qIP-1a* showed *R*^*2*^ values of up to 29.12%, they should be considered major M-QTLs. On LG10, two sets of M-QTLs, *qDS-10a* and *qDInt-10b*, and *qDS-10b* and *qDInt-10c* respectively, were consistently present in AFT and FT3 analyses. Also, over the distal region of LG10, the major M-QTL *qDS-10d*, which was associated with GLP_4 marker, showed the highest *R*^*2*^ value (29.65%) across the study. Finally, *qDI-15a* on LG15 showed moderate *R*^*2*^ values and was consistently detected across three field trials over the candidate gene clusters composed by HeAn_R_534.1, HeAn_R_174.2, HeAn_C_11434.1, and HeAn_C_12025.1.

Figs 1–4 show all M-QTLs that co-located with QTLs from earlier maps in bold. This is the first report on SHR-related QTLs on LG11 and LG15; consequently, the seven M-QTLs identified in this work for these LGs, *qDInt-11a*, *qDInt-11b*, *qDInt-15a*, *qDInt-15b*, *qIP-15a*, *qDI-15a*, *qIP-15b*, are new.

### Epistatic QTLs and environmental interactions for SHR-related phenotypic variable

Two-locus analysis detected 13 Ep-QTLs involved in seven significant digenic epistatic interactions on LG2, LG3, LG7, LG8, LG9, LG10, LG11, LG15, LG16 and LG17 (Figs 1–4 and Table 5, arrows points from QTL_*i*_ to QTL_*j*_).

**Table 5:** Parameters associated with epistatic QTLs involved in additive-by-additive interactions detected in BMP using QTLNetwork.

Trait	QTL_*i*_	LG	Marker^a^	Position on LG (cM)	Confidence interval (cM)	QTL_*j*_	LG	Marker^a^	Position on LG (cM)	Confidence interval (cM)	aa^b^	h^2^aa (%)^c^	QTL × QTL × Environment interactions		
													Field Trial^d^	aae^e^	h^2^aae (%)^f^
**DI**															
	*qDI-8a’*	8	ORS894_2	173.00	4.80	*qDI-10a’*	10	E33M60_2	204.50	4.80	-0.02	0.72	-	-	-
**DS**															
	*qDS-2a’*	2	HeAn_R_174.1	35.50	6.90	*qDS-15a’*	15	E38M48_4	99.40	4.20	0.04	2.29	FT2, FT3	-0.04 to 0.06	1.15 to 3.26
	*qDS-7a’*	7	E32M49_11	79.60	4.70	*qDS-9a’*	9	E33M60_5	156.80	4.60	0.05	4.57	FT2	0.05	2.16
	*qDS-7b’*	7	E40M62_21	87.90	3.00	*qDS-11a’*	11	HeAn_C_5453.1	24.50	6.00	0.04	3.18	-	-	-
**DInt**															
	*qDInt-3a’*	3	HA1770	121.90	7.50	*qDInt-17a’*	17	SSL87	48.60	11.60	0.04	9.80	-	-	-
**IP**															
	*qIP-16a’*	16	HeAn_R_126.2	13.10	3.40	*qIP-17a’*	17	E38M50_21	113.50	8.30	0.46	6.25	-	-	-
	*qIP-16a’*	16	HeAn_R_126.2	13.10	3.40	*qIP-17b’*	17	TC26323	130.30	8.00	0.45	4.69	FT2	-0.80	1.94

^a^Marker defining QTL, closer to the highest LOD value.

^b^Additive by additive interaction between *loci i* and *j*.

^c^Percentages of the phenotypic variations explained by additive by additive interactions.

^d^Field Trial = Field trial in which Q × E was detected for the particular QTL. FT1 = Balcarce October 2009, FT2 = Balcarce December 2011, FT3 = Balcarce December 2012, FT4 = Balcarce December 2013, AFT = across field trials, pooled data from all the trials.

^e^Effect of the epistasis × environment interactions.

^f^Percentage of the phenotypic variations explained by the epistasis × environment interactions.

DI = Disease Incidence

DS = Disease Severity

DInt = Disease Intensity

IP = Incubation Period

None of the Ep-QTLs involved in Q × Q interactions showed main-effects. Epistasis contributed 0.72% (DI), 10.04% (DS), 9.8% (DInt) and 10.94% (IP) of the total PVE. Mostly for all Ep-QTLs, recombinant two-locus combinations enhanced resistance. However, for DI only one parental two-locus combination enhanced resistance.

Finally, we detected three QTL × QTL × Environment interactions for DS and IP and these interactions involved FT2 and FT3.

## Discussion

Screening for disease resistance in inbred lines and commercial hybrids is performed periodically as part of the ongoing sunflower breeding programs and annual variety trials. Assisted inoculation with ascospores, like the one used in this work, is preferable since natural infections are affected by the presence of sclerotia in the soil and weather conditions, which vary between years and regions [29,59]. Assisted inoculation is also highly reproducible and suited for revealing the variability in disease resistance response in sunflower [60]. We performed the inoculation of each capitula with 2,500 ascospores and analyzed four phenotypic variables, DI, DS, DInt and IP to differentiate between susceptible and highly resistant sunflower RILs. In this study, the amount of ascospores used in assisted inoculation was 10-fold less than in Castaño *et al*. [61], Van Becelaere and Miller [29] and Vear *et al*. [62] and the levels of disease varied between 0 to 100%.

SHR disease develops in stages, starting with the infection during flowering, followed by mycelium invasion in the parenchyma tissues during the grain-filling, and ending with sclerotia formation at maturity [63]. The phenotypic variables measured in this work allow the evaluation of the RIL’s performance during the different stages of the disease development. DI and IP measure the resistance to pathogen penetration and the delay in the initial mycelium growth. Whereas, DS and DInt account for disease resistance in intermediate and final phases of SHR development [64].

We found a significant correlation between DS and DInt (r = 0.8). It was expected because both variables consider the proportion of infected area in their assessments. Despite the relationship between these indicators, we still consider both as separate variables because the likelihood of finding different QTLs for SHR resistance was increased when both variables were included in the analysis. All other variables showed a moderate to low correlation between them. These results suggest that different genetic bases (partially shared among variables) are involved in SHR resistance, as proposed by Bioley *et al*. [65].

The high sensitivity of genotype response to environmental conditions became apparent in the present work where DI and DInt means for FT2 differed from those of the other field trials. Indeed, the FT2 showed the highest average daily temperature during the RILs inoculation period (23.7°C vs. 21.8°C average for the others FTs). The inhibitory effect of higher temperatures on fungal pathogenicity is reflected on the positive skewness, kurtosis and CV values registered during FT2 (Table 1). Filippi *et al*. [30], who studied SHR resistance with association mapping in the same location, also observed that the DI and DS values were below the overall mean in an assay conducted in 2011/2012. In agreement with our findings, Abawi and Grogan [66] found a negative correlation between temperature and SHR disease levels. They reported that the average temperatures for proper growth of the Sclerotinia mycelia were between 16 and 22°C. Similarly, Vear *et al*. [67] described a lower level of attack by ascospores in a variety field trial conducted in Clermont-Ferrand in 2003 than in previous trials in the same location. In their study, temperatures ranged from 25°C at night to 40°C at day time.

Moreover, inoculation dates also seem to affect the disease response. For instance, FT1 sown in October showed longer IP (and consequently lower disease levels at 21 DPIs) than the other three FTs, which were sown in December of each year.

In this study, we applied the mixed model approach to work with complex data. The effect of different inoculation dates on disease response was solved by including this variable as a random effect in the statistical models. Also, the adjusted means calculated for the four phenotypic variables, allowed us to test the genotype-by-environment interactions G × E, i.e. the differential genotypic response to different environments.

We observed a broad range of *h*^*2*^ values, ranging from 1.86% to 59.9%. DI and DInt displayed the lowest *h*^*2*^ values during FT2. This finding was expected due to the unfavorable environmental conditions for the development of the disease during this trial. These values are comparable to those obtained by Filippi *et al*. [30] using the same approach. The moderate *h*^*2*^ values suggest that marker assisted selection, using either the AM or the BMP approach, may be the method of choice when breeding for resistance to SHR, instead of conventional phenotypic selection.

The PAC2 × RHA266 BMP is a reference population used in many QTL studies for different traits [68–71]. Despite that, the available reference maps for this population are based on AFLP and SSR markers. Indeed, the use of new generation genotyping platforms to saturate the PAC2 × RHA266 map has not been reported yet.

By using a custom-made SOPA (Illumina), we incorporated 61 new SNP markers into an already existing genetic map, thus giving an average of one marker per each 4.45 cM. This map updates the previous linkage map published by Talia *et al*. [10], built using 94 RILs from the same population. In comparison, Flores Berrios *et al*. [71], Bert *et al*. [21] and Rönicke *et al*. [72] described slightly shorter maps with sizes of 2,558 cM, 2,318 cM, and 2,273.5 cM, respectively. Extreme caution must be taken when comparing maps, because different software and the adjustment of various parameters (e.g. LOD, recombination frequency) can influence the length of the map. Furthermore, this variation can result from marker clusters or different distribution patterns among types of markers, the effects of distorted segregation markers and population sizes [73,74].

We observed a uniform distribution of the added SNP markers in the sunflower genome. Eight markers, HeAn_R_149.1, HeAn_C_10204.1, HeAN_R_RGEF_A1.2, HeAn_C_4023.2, HeAn_C_11741.1, HeAn_R_204.1, HeAn_C_12910.1 and HeAn_C_5433.3, mapped on the telomeric positions on LG2, LG4, LG9, LG11 and LG17. We obtained good marker saturation with only three gaps greater than 25 cM located on LG9 and LG17.

The large number of detected main effects QTLs (36) supports a polygenic inheritance and a substantial influence of environmental factors on resistance to SHR. Moderate additive effects were detected for alleles for most of the evaluated variables (Tables 3 and 4). For all the detected M-QTLs, the *R*^*2*^ and additive effects values found in were similar to those reported by Bert *et al*. [21,75]*et al*., Rönicke *et al*. [72] and Yue *et al*. [20].

Some RILs produced a lower SHR disease level when compared to their parents, while others produced higher levels. This finding suggests transgressive segregation for resistance in this cross. This was supported by QTL mapping since the alleles conferring increased resistance against SHR were originated from the partially resistant parental line (P_R,_ PAC2) for the 85% of the detected M-QTLs. Several researchers have previously reported trangressive segregation in sunflower for partial resistance to *S*. *sclerotiorum* stem rot [76,77] and SHR [22].

To analyze the genetic basis of SHR resistance we assessed the co-localization of the detected QTLs for the four phenotypic variables across FTs, as well as conserved collinearity regions among reported QTLs contributing to resistance to different forms of Sclerotinia resistance in sunflower. Identification of overlapping QTLs could increase the efficiency of marker-assisted selection and enhance genetic progress [78]. In this work, we detected clustered M-QTLs on LG1 (eight), LG10 (five) and LG15 (eight). M-QTLs for maximum incidence was consistently present on the distal region of LG15 across field trials, suggesting that their expression may not be sensitive to environmental conditions. No previous QTLs for SHR resistance seemed to be on LG15. However, Micic *et al*. [79] found statistically significant clustered QTLs for midstalk-rot accounting for the speed of fungal growth and size of the lesion in both, leaf and stem, in this linkage group. The QTLs for fungal’s growth speed described by Micic *et al*. [79] co-locate with the genomic region that we studied on LG1. Similarly, most M-QTLs on LG10 could be associated with the section harboring the branching gene (*b1*) close to the SSR marker ORS437 [22]. In agreement with previous studies carried out on PAC2, this branched line may have a fixed favorable allele for Sclerotinia resistance near to, or in the same zone, as that of the recessive apical branching gene *b1* [22,80]. Bert *et al*. [75] and Yue *et al*. [20] identified other QTLs in the same chromosomal region. According to our results, M-QTLs located on LG1, LG10 and LG15 have good reproducibility, high *R*^*2*^ values and seemed to be involved with common resistance responses (Figs 1–4, Tables 3 and 4). Thus, these genomic regions must be considered as candidate regions for breeding programs.

Our results support the putative role of candidate regions on LG1, LG10 and LG15 during the SHR disease. However, M-QTLs were also detected in agreement with another authors on LG2 [20], LG8 [75], LG10 [19,20,72,75], LG11 [79], LG15 [21] and LG16 [79]. Through an AM approach, we also identified one QTL, supported by the marker HA1848 on LG7 significantly associated with resistance to SHR [81]. The genomic region of this QTL is large (3.3 and 7.7 cM at both sides of the marker HA1848) and consequently, makes it difficult to determine which genes are involved in this resistance. However, HA1848 is a promising marker for use in breeding programs since both approaches have validated it and also because it was located on LG7, which was already mentioned in several QTL mapping papers on for *S*. *sclerotiorum* resistance, including SHR in sunflower [20,21,75].

When we analyzed the Ep-QTLs, none of them had main effects. However, previous researches have reported the identification of *loci* with no main effect but with the ability to influence a variable through their interactions with other *loci*. This suggests that epistatic interactions between minor Ep-QTLs may play a significant role in enhancing overall resistance [82]. No hub regions associated with resistance variables were identified.

We identified a low number of both QTL × Environment interactions and QTL × QTL × Environment interactions. This finding could either be explained because parental QTL alleles interacting with the environment do not differ, or because these characters are stable and independent of environmental influence. This result is desirable since the use of QTLs not involved in interactions for molecular-assisted selection (MAS) guarantees a better performance of the breeding materials in different environments.

This study constitutes the most extensive report of QTLs for resistance to SHR up to the present. The relatively moderate number of RILs used in our study may have had an adverse influence on the accuracy of the calculated QTL effects and the ability to detect QTLs with small effects [83]. However, this was compensated by the higher precision of the phenotypic data estimation and the use of a new saturated map with 635 markers. In this regard, while the development of closely linked markers and the validation of most of the detected QTLs is required, major candidate regions on LG1, LG10 and LG15 were also identified in different independent studies. Thus, these regions are candidates to breeding programs through MAS. MAS involve a limited number of QTLs to be transferred from one genetic background to another (therefore pyramiding all minor QTLs is not possible). Alternative approaches like marker-assisted recurrent selection (MARS) or genome-wide selection (GWS) allow selection for several QTLs with small effects [84].

## Conclusion

The wide distribution of the QTLs identified here confirmed the vast and complex genetic basis of sunflower resistance to SHR. This work deepens the knowledge of genomic regions associated with SHR resistance in sunflower and highlights the importance of the reduced groups of QTLs on LG1, LG10, and LG15. These genomic intervals are presented as candidates. Progress could be achieved by applying different approaches in genomics and genetics, both to locate functional components responsible for the expression of resistance and to contribute to the understanding of the complexity of resistance. The improved genetic map includes 61 SNP markers over candidate genes providing strong evidence of the power of biparental populations in the genetic study of disease resistance and establish an appropriate platform for detecting QTLs. Finally, the QTL associated markers described here could be transferred to breeding programs to accelerate the process of obtaining genotypes with better performance against SHR.

## Supporting information

S1 Table Pearson correlation coefficient among four traits in BMP. Pearson correlation coefficient (r) and their correspondent *p*-value significance over the main diagonal.
(XLSX)

S2 Table Source of the mapped SNPs. Database and Accession Number from which polymorphisms were derived are provided.
(XLSX)
